# Multi-modal strain mapping of steel crack tips with micrometre spatial resolution

**DOI:** 10.1107/S1600577525008100

**Published:** 2025-10-16

**Authors:** Ahmar Khaliq, Felix Wittwer, Anna Wildeis, Markus Hartmann, Matthias Thimm, Robert Brandt, Dennis Brueckner, Jan Garrevoet, Gerald Falkenberg, Peter Modregger

**Affiliations:** a Department of Physics University of Siegen 57068Siegen Germany; b Center for X-ray and Nano Science CXNS Deutsches Elektronen-Synchrotron DESY 22607Hamburg Germany; c Department of Mechanical Engineering University of Siegen 57068Siegen Germany; d Deutsches Elektronen-Synchrotron DESY 22607Hamburg Germany; Advanced Photon Source, USA

**Keywords:** crack propagation, strain, martensite, X-ray diffraction, shot peening

## Abstract

Due to their superior fatigue strength, martensitic steels are the material of choice for high cyclic loading applications such as coil springs. However, crack propagation is influenced by residual stresses and their interaction is poorly understood. In fact, linear elastic fracture mechanics predicts unphysical singularities in the strain around the crack tip. In this study, we have combined synchrotron-based X-ray diffraction, X-ray fluorescence and optical microscopy to map the factual strain fields around crack tips with micrometre spatial resolution. X-ray fluorescence and optical images were co-registered to locate the crack in the X-ray diffraction maps. Observed crystal recovery close to cracks confirmed that the diffraction signal originates at least in part from the cracks. The retrieved local strain field around the crack was further improved by averaging information over carefully selected diffraction peaks. This procedure provided strain maps around crack tips with a spatial resolution of about 1 µm and may enable heuristic predictions of further crack growth.

## Abstract 2

Multi-modal measurements combining X-ray diffraction, X-ray fluorescence and optical microscopy allow dynamic crystal recovery effects around martensitic steel crack tips to be studied. The measured strain field around the crack tip shows a significant departure from the predictions of linear elastic fracture mechanics.

## 1. Introduction

Martensitic steel is widely used in engineering applications due to its high strength, high wear resistance (Wu *et al.*, 2023 ▸) and favorable fatigue properties (Heshmati *et al.*, 2023 ▸; Sun *et al.*, 2020 ▸; Zhou *et al.*, 2024 ▸). The transformation from austenite to martensite occurs through a diffusionless process (Shewmon, 1969 ▸; Kelly, 2012 ▸), resulting in a highly distorted lattice structure that enhances its mechanical performance (Hutchinson *et al.*, 2022 ▸). Residual stresses and microstructural heterogeneities play a crucial role in the mechanical behavior of martensitic steels, for example influencing fatigue crack initiation and early propagation (Li *et al.*, 2025 ▸; Kim *et al.*, 2022 ▸). This can occur intergranularly along grain boundaries and transgranularly through the grains (Wildeis *et al.*, 2022 ▸). However, the interaction between residual stresses and crack propagation remains poorly understood in these steels.

Crack propagation is studied within the field of fracture mechanics, which provides a fundamental approach for predicting crack growth and material failure under various loading conditions (Anderson & Anderson, 2005 ▸). A prominent approach is linear elastic fracture mechanics (LEFM), which assumes linear elasticity (Anderson & Anderson, 2005 ▸; Broberg, 1999 ▸). According to LEFM, a crack can experience three modes of loading. Mode I is the opening mode, which occurs when the load is applied perpendicular to the crack plane (mode I is usually a result of tensile stresses and is primarily accountable for crack growth). Mode II is the sliding mode – the load acts as an in-plane shear load where the loading makes one crack face slide relative to the other. Mode III is the tearing mode, where out-of-plane shear stresses result in displacements parallel to the crack tip edge (Jensen, 2015 ▸).

LEFM is most applicable to cracks, particularly under mode I loading. In this case, the stress component in the crack propagation direction near the crack tip is described by (Anderson & Anderson, 2005 ▸)
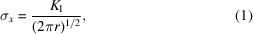
where *K*_I_ is the mode I stress intensity factor and *r* is the distance from the crack tip. At *r* = 0 this equation predicts a singularity (*i.e.* infinite stress), which is unphysical. A similar situation holds true for the strain around a crack tip.

Furthermore, martensite was chosen as the material system as its complex mesoscopic structure interacts with the stress field during crack propagation. For example, this leads to an experimentally more challenging tortuous crack growth over linear crack growth as predicted by LEFM. However, the stress field versus crystallographic structure interaction is poorly understood, which leads to the inability to provide fracture-mechanical proof of fatigue strength for structural components (Wildeis *et al.*, 2022 ▸).

The simultaneous acquisition of synchrotron-based X-ray diffraction (XRD) and X-ray fluorescence (XRF) has proven to be a powerful tool for enhancing the understanding of material properties on the micrometre scale. Examples include the interplay of local strain and material composition on the performance of thin film solar cells (Ulvestad *et al.*, 2019 ▸; Calvo-Almazan *et al.*, 2019 ▸), the characterization of extraterrestrial samples (Lanzirotti *et al.*, 2024 ▸) or the application in chemical analysis (Su *et al.*, 2024 ▸).

In this study, we have combined micro-XRD (for strain measurements), XRF (for crack localization via self-absorption-based contrast) and optical microscopy (for high-resolution imaging of cracks) to map strain fields around crack tips with micrometre resolution in martensitic, real-world tensile testing samples. This was done for three samples, two shot-peened samples and one un-modified sample. *Ex situ* transmission electron microscopy (TEM) or high-resolution TEM (HRTEM) are beneficial for atomic-scale strain measurements but were excluded due to the destructive preparation that alters macroscopic residual strains and stresses (Hÿtch & Minor, 2014 ▸).

## 2. Materials and methods

The following sections detail the methods employed in this study, covering sample preparation, optical microscopy for crack monitoring, and synchrotron XRD and XRF experiments at PETRA III, DESY.

### 2.1. Sample preparation

The specimens were prepared from high-strength martensitic spring steel SAE 9254 (DIN/EN: 54SiCr6). The steel was austenitized under vacuum at 1080°C for 100 min, quenched with nitrogen and tempered at 400°C for 1 h in argon to form a martensitic structure. The resulting martensitic structure forms as lamellae that are 0.2–0.5 µm thick (Maki, 2012 ▸). Following heat treatment, three samples were machined from 12 mm-diameter wire rods via electric discharge machining, featuring a gauge section of 10 mm length, 5 mm width and 2 mm thickness to promote surface crack initiation. Two specimens were shot-peened (Guagliano, 2001 ▸) with an Almen intensity of 0.16 mm using a pneumatic system equipped with dual nozzles, operated at 1.5 bar pressure, and steel shots with a diameter of 0.4 mm (700 HV, G3 per VDFI 8001). This ensured full surface coverage and introduction of in-plane compressive residual stresses estimated at 900 MPa (Wildeis *et al.*, 2021 ▸; Wildeis *et al.*, 2022 ▸). The third specimen remained unpeened.

To study crack behavior, cracks were initiated by applying uniaxial cyclic loading at a stress ratio of *R* = −1 (*i.e.* the ratio of minimum to maximum stress), a frequency of 10 Hz, and stress amplitudes varied between 550 MPa and 680 MPa, corresponding to high cycle fatigue conditions. Slip bands formed at prior austenite grain boundaries, acting as crack initiation sites, with shot-peened specimens showing delayed crack growth due to residual stresses (Wildeis *et al.*, 2021 ▸; Wildeis *et al.*, 2022 ▸).

### 2.2. Optical microscopy

Optical microscopy was used to track crack initiation and early propagation across the specimens. Surface preparation involved progressive grinding with SiC paper up to grit 4000, followed by a final polish with a colloidal silicon suspension of 0.25 µm grain size to yield a smooth, reflective finish. A confocal laser microscope (Olympus LEXT OLS4000) was employed to acquire detailed images of the specimen surfaces at scheduled intervals during fatigue testing. These images allowed for the measurement of crack lengths and the evaluation of crack density, offering insights into the progression of fatigue damage across both treated and untreated conditions (Wildeis *et al.*, 2021 ▸; Wildeis *et al.*, 2022 ▸).

### 2.3. Synchrotron-radiation experiment

**Figure 1:**
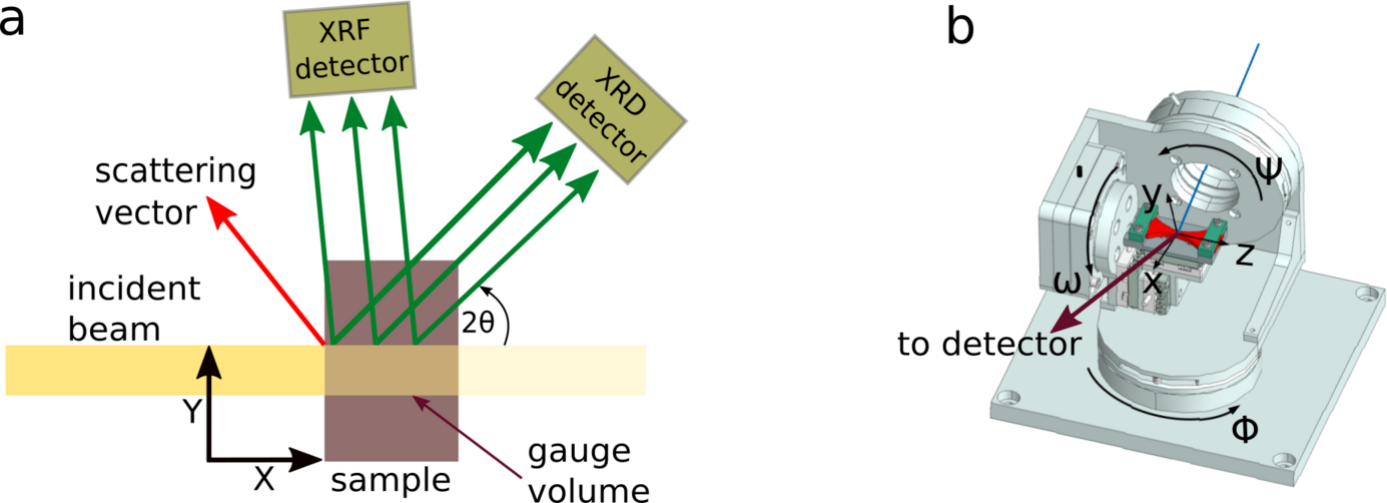
(*a*) Schematic of the experimental setup, top view. (*b*) Goniometer configuration used for sample positioning.

The goal of this study was to demonstrate the possibility of measuring the strain field around crack tips with micrometre spatial resolution for samples that are compatible with fatigue testing series. The synchrotron-radiation experiment was conducted at the P06 beamline of PETRA III at DESY, Hamburg (Falkenberg *et al.*, 2020 ▸), featuring a unique combination of small beam sizes at high photon energies, which are essential to achieve the stated goal. A sketch of the utilized setup is shown in Fig. 1 ▸(*a*), which was also described by Chakrabarti *et al.* (2022 ▸). The setup is a hybrid between reflection and transmission geometry, with the beam entering on one surface and exiting at an orthogonal surface. A monochromatic X-ray beam of 35 keV was selected by a Si (111) double-crystal monochromator. The beam was focused using compound refractive lenses (CRLs), achieving a beam size of *h* × *v* = 0.9 µm × 0.4 µm, with a penetration depth of 276 µm, resulting in a gauge volume of 99.4 µm^3^. The sample was mounted on a six-axis goniometer, which allowed precise alignment and positioning during measurements [see Fig. 1 ▸(*b*)]. The utilized coordinate system is consistent with the work of Chakrabarti *et al.* (2022 ▸). The diffraction signal was recorded using an XRD detector [see Fig. 1 ▸(*a*)] with 55 µm pixel size, positioned approximately 1.0 m downstream of the sample and horizontally inclined at 25° relative to the incident beam. Simultaneously, XRF data were collected using a silicon drift detector (Hitachi High-Tech), positioned slightly offset from 90° relative to the incident beam for effective fluorescence detection. Two scans were performed for each sample: one overview scan, covering the entire crack with a step size of 10 µm and an exposure time of 0.2 s per point, and another focused on the crack tip with a step size of 1 µm and the same exposure time of 0.2 s.

## 3. Data analysis

In this section, we focus on processing XRD and XRF data through key steps: collecting diffraction patterns, fitting peaks, locating cracks, analyzing the effects of crystal recovery, quantifying strain and mapping the strain field at the crack tip in martensitic steel samples.

**Figure 2:**
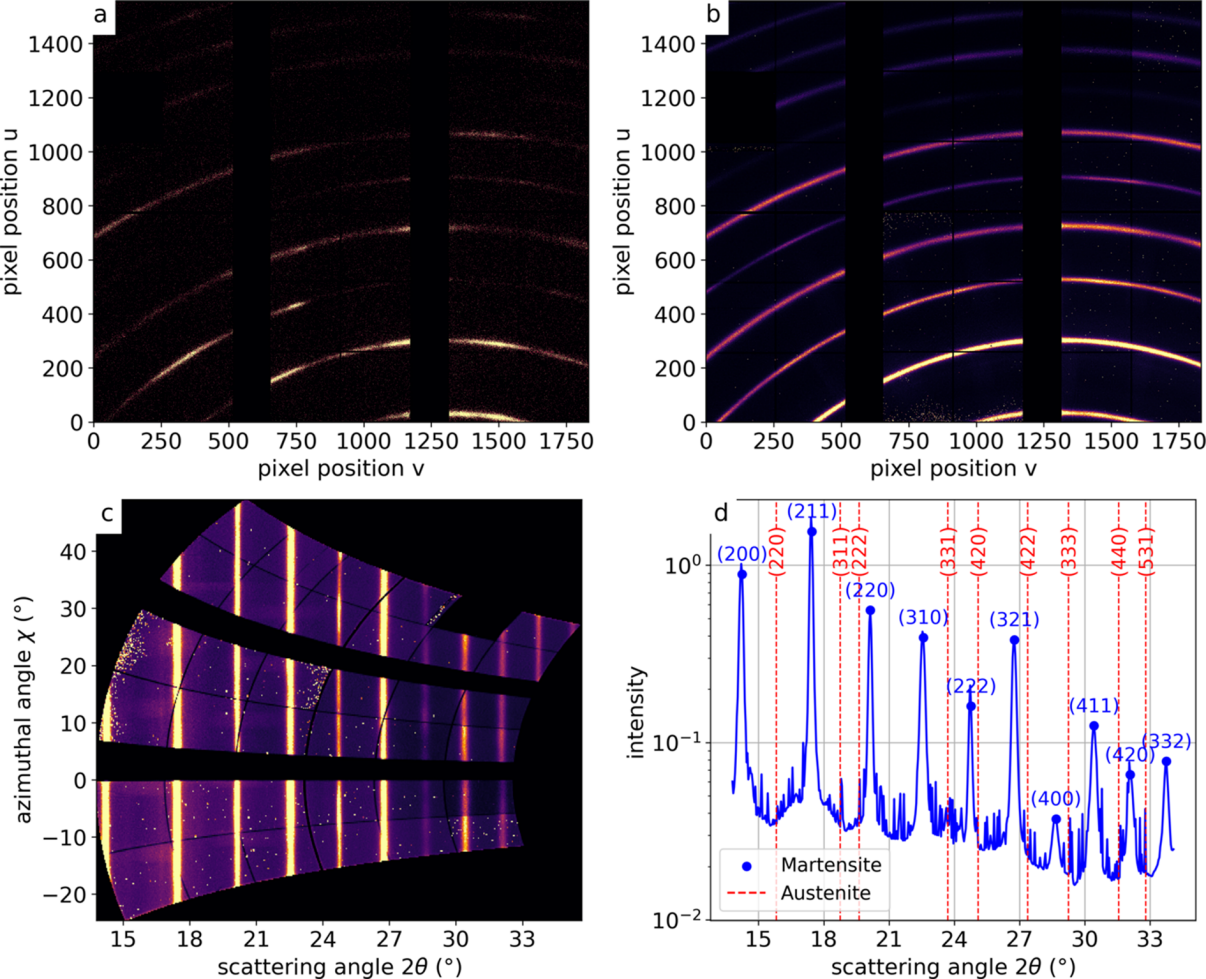
(*a*) A diffraction pattern of a single scan point of a martensitic steel sample. (*b*) Diffraction pattern summed over all 14091 scan points. (*c*) Transformation to polar coordinates of the summed diffraction pattern via caking. (*d*) Resulting 1D integration with the 2θ positions of martensite and austenite indicated.

The experimental geometry was calibrated using the *pyFAI* calibration routine (Ashiotis *et al.*, 2015 ▸) with lanthanum hexaboride (LaB_6_, NIST SRM 660c) (Black *et al.*, 2020 ▸) as a diffraction standard. The calibration was performed with the LaB_6_ powder sample mounted on the goniometer stage. In this setup, the relevant scattering geometry was given by the relative position of the detector and the illuminated part of the sample. The latter was defined by the position of the fixed X-ray beam itself, which did not change during a scan. The sample was scanned through the X-ray beam, which guarantees that the calibrant and the measured samples had identical scattering geometries. Diffraction rings from LaB_6_ were recorded and fitted using the *pyFAI* software to determine the detector geometry (sample-to-detector distance, beam center coordinates and detector tilts). For automatic peak detection diffraction patterns from all scan points [see Fig. 2 ▸(*a*)] were summed [see Fig. 2 ▸(*b*)]. Some crystallographic texture is visible in the diffraction pattern of a single scan point as a variation of intensity along a given ring [see Fig. 2 ▸(*a*)]. Apparently, the coherent domains are much smaller than the gauge volume utilized. In the following, we will neglect crystallographic texture and treat the signal as a powder X-ray diffraction signal.

Individual diffraction patterns were transformed from Cartesian coordinates (*u*, *v*) to polar coordinates (2θ, χ) via a caking process (Kieffer & Karkoulis, 2013 ▸), with an example shown in Fig. 2 ▸(*c*). Here, 2θ is the scattering angle between the incident and the diffracted beam, χ is the azimuthal angle around the beam axis [see Fig. 2 ▸(*c*)]. The gaps in the patterns in Fig. 2 ▸(*c*) arise from inactive regions between detector modules, appearing as zeros in the raw data. These were masked out during azimuthal integration, ensuring only valid pixels contribute to the profiles and peak fitting. Subsequent azimuthal integration, performed over a χ range from −20° to 20°, yielded the diffraction signal as a function of 2θ. As shown in Fig. 2 ▸(*d*), the first ten martensitic diffraction peaks, ranging from [200] to [332], were identified.

**Figure 3:**
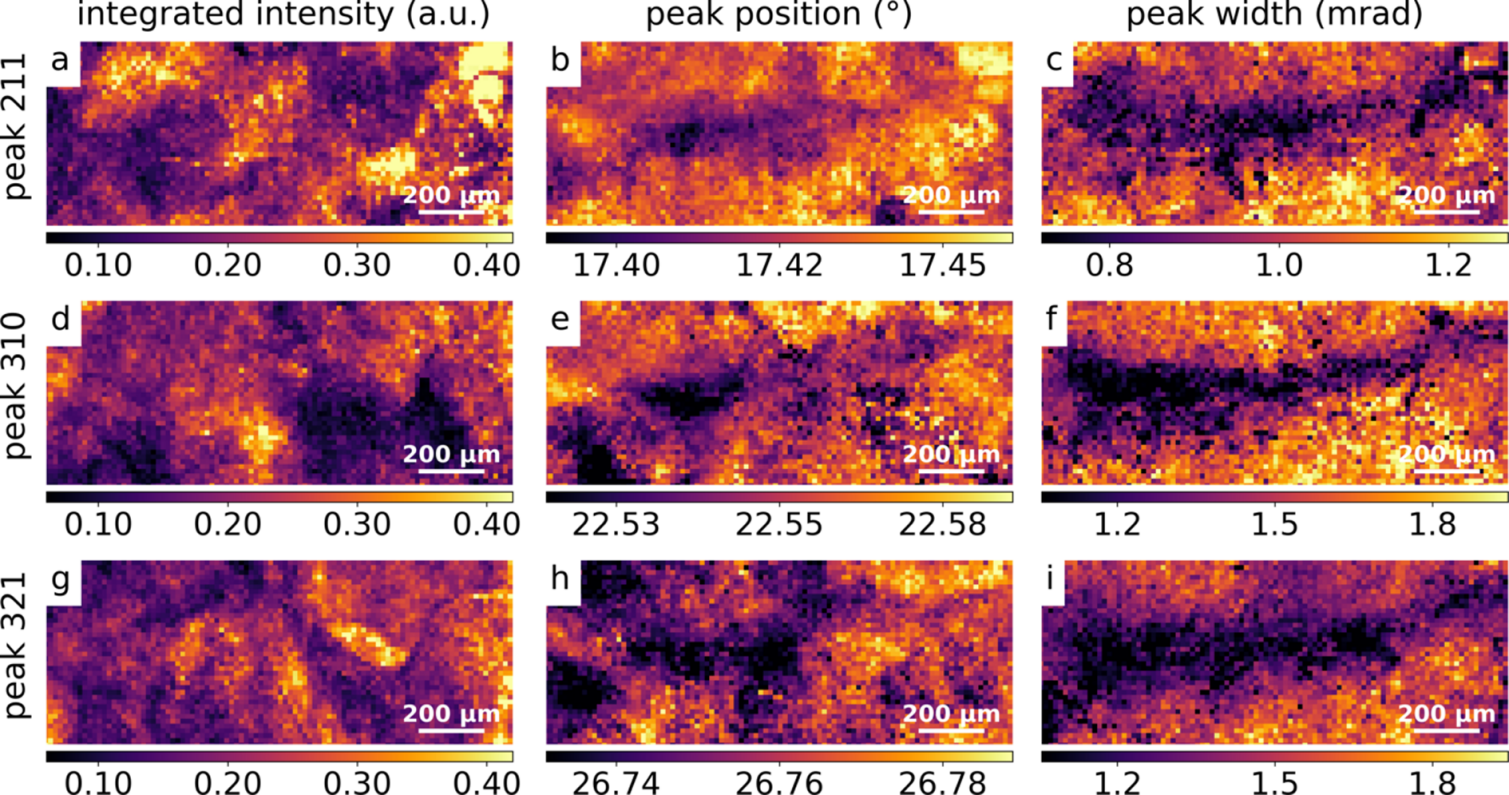
Maps from the overview scan showing integrated intensity (left column), peak position (middle column) and peak width (right column) for peaks [211], [310] and [321]. Rows correspond, respectively, to: peak [211] in (*a*–*c*), peak [310] in (*d*–*f*) and peak [321] in (*g*–*i*).

For the determination of the peak height *H*, the angular peak position μ, the angular peak width σ and the background *C* of all occurring diffraction peaks, the azimuthal integrated intensities were fitted to a Gaussian distribution given by 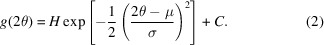
To study local variations, using the fitted Gaussian parameters, maps for peak height, peak position and peak width were computed for each scan point of each peak. The integrated intensity *I*_int_ was obtained using *I*_int_ = *H*(2π)^1/2^σ. Maps of the integrated intensity, peak position and peak width for the overview scan were obtained for all peaks; maps for peaks [211], [310] and [321] are shown as examples in Figs. 3 ▸(*a*)–3 ▸(*i*). The lattice parameter of unstrained martensitic steel was estimated by the observed, averaged lattice parameter *a*_0_. The latter was retrieved from ten peaks in the diffraction pattern averaged over an entire scan. The result was *a*_0_ = 2.866 (4) Å, which is in agreement with published values (Xiao *et al.*, 1995 ▸).

**Figure 4:**
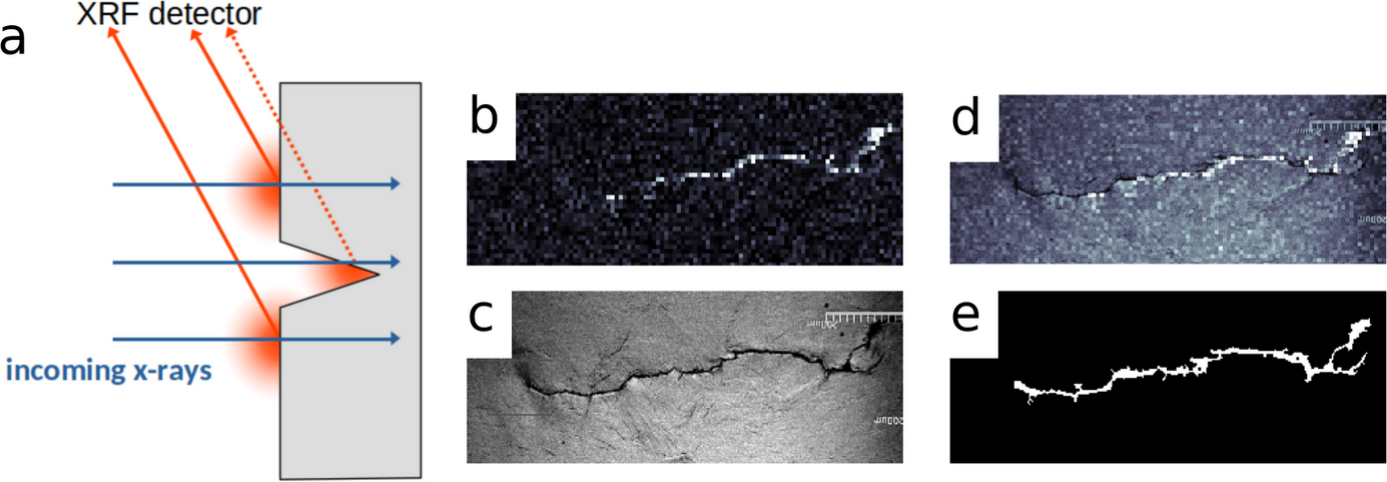
Process of crack localization using XRF and microscopy: (*a*) schematic of the XRF process, (*b*) XRF image, (*c*) optical microscope image, (*d*) registered XRF and microscopy images, (*e*) crack segmentation.

Since cracks are not directly visible in the XRD maps, the cracks were located using the self-absorption of fluorescent X-rays within the material, which blocks the XRF signals and creates highlighted and shadowed areas in the XRF image [similar to scanning electron microscopy (Baba-Kishi, 1994 ▸)], as shown in Fig. 4 ▸(*a*). The resulting XRF image is shown in Fig. 4 ▸(*b*). However, due to the limited spatial resolution and contrast, crack tip segmentation directly from XRF data was challenging. To achieve accurate crack segmentation, optical microscope images [see Fig. 4 ▸(*c*)] were acquired and registered onto the summed XRF image (total XRF), enabling crack localization, as depicted in Fig. 4 ▸(*d*). The resulting crack silhouette is shown in Fig. 4 ▸(*e*).

**Figure 5:**
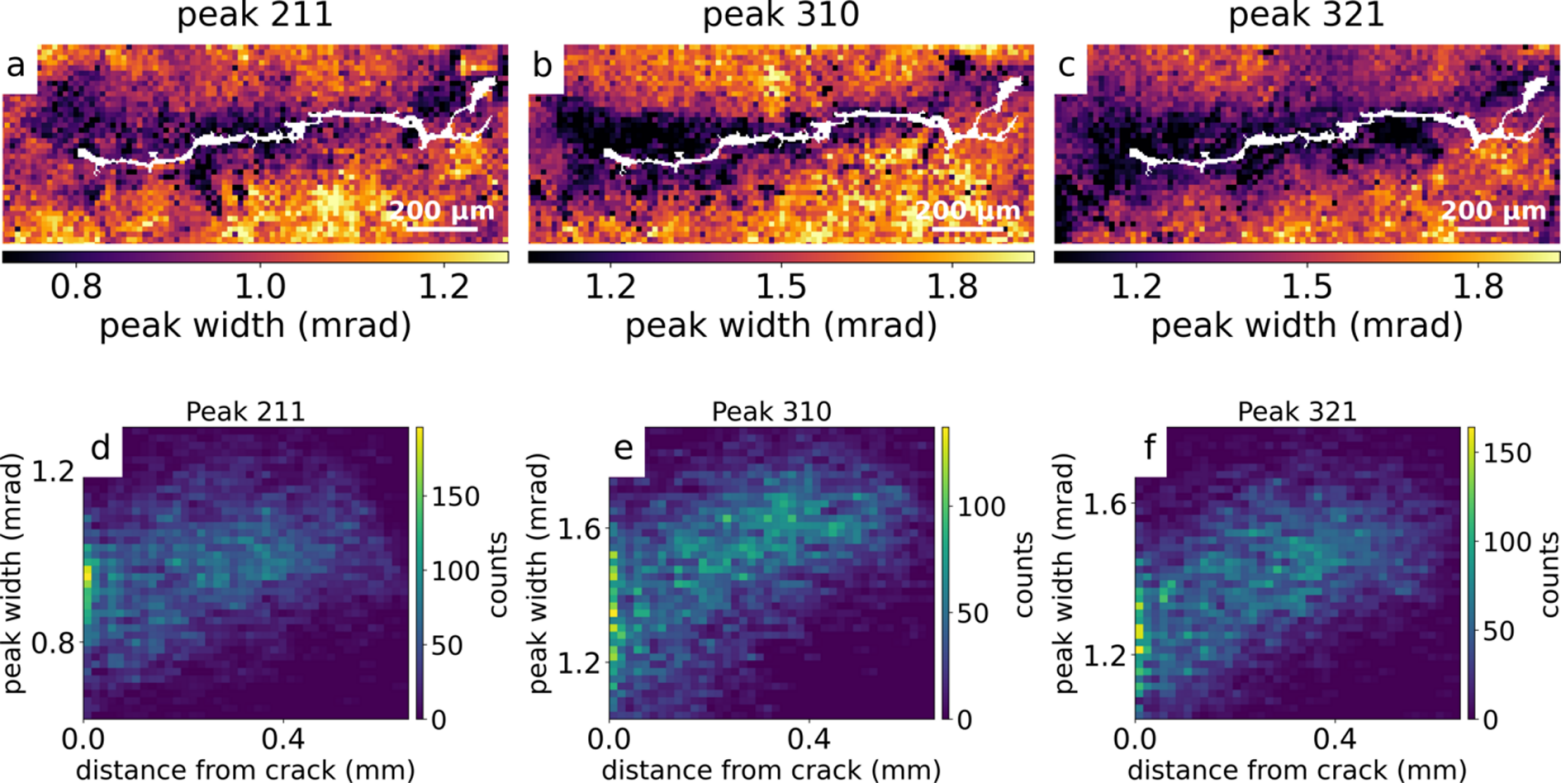
Peak width maps with crack overlay and their corresponding 2D histograms of peak width versus crack distance: (*a*+*d*), (*b*+*e*) and (*c*+*f*). Here, we observe effects of crystal recovery up to 300 µm from the crack for the peaks [211], [310] and [321], respectively.

The crack silhouette [see Fig. 4 ▸(*e*)] was overlaid onto the XRD maps to investigate variations near the crack. As an example, three peak width maps with the crack overlay are shown in Figs. 5 ▸(*a*)–5 ▸(*c*). All three maps show a decrease in peak width close to the crack. This is verified by the 2D histogram of the peak widths versus crack distance (defined as the shortest length from each pixel to the closest crack pixel), which shows an obvious trend up to 300 µm from the crack [see Figs. 5 ▸(*d*)–5 ▸(*f*)]. According to the Scherrer equation (Williamson & Hall, 1953 ▸), this indicates an increased crystallite size close to the crack. This change in crystallite size could be caused by recrystallization; however, previous measurements showed no evidence of recrystallization in the vicinity of the crack (Wang *et al.*, 2024 ▸; Wildeis *et al.*, 2022 ▸). Instead, it is likely caused by dynamic recovery which reduces local microstrain and dislocation density and thereby decreases peak width (Wang *et al.*, 2024 ▸; Miao *et al.*, 2017 ▸). Furthermore, this confirms that at least some diffraction information originated from the vicinity of the crack.

The strain along the scattering vector ɛ_*q*_ was determined using the differential Bragg equation (Hart, 1969 ▸),
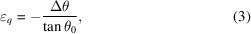
where θ_0_ is the average peak position (corresponding to *a*_0_) and Δθ = θ_measured_ − θ_0_ represents the peak shift at each scan point. Assuming that the sensitivity of retrieved strain values was limited by photon shot noise (*i.e.* negligible drift), we have used the approach laid out by Modregger *et al.* (2025 ▸) and the above equation to estimate a precision of *u*(ɛ_*q*_) = 1.5 × 10^−5^ for the [321] reflection. Similar values hold true for the other reflections used below.

**Figure 6:**
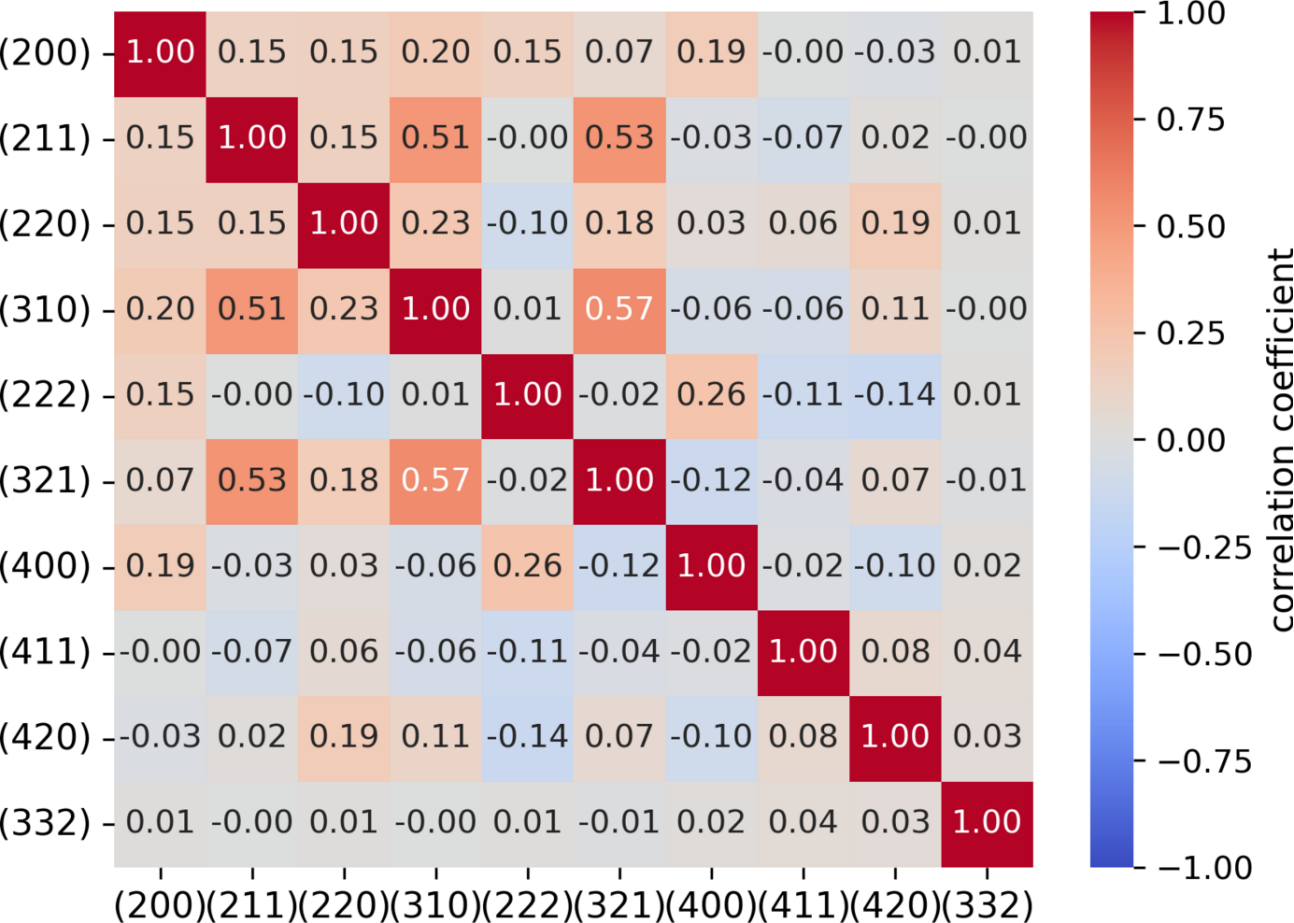
Heatmap of correlation coefficients of peak width maps, which we used to identify the following highly correlated peaks: [211], [310] and [321].

To improve the contribution from the crack vicinity, we combined the strain information from several different diffraction peaks. To this end, we have calculated the pairwise correlation coefficient between peak width maps of different peaks. The corresponding heatmap is shown in Fig. 6 ▸. Here, we have selected three peaks with the triple (*i.e.* [211], [310] and [321]) of largest correlation coefficients (*r* = 0.51–0.57), which are – not coincidentally – the peaks with highest multiplicity.

The projection of the 2D strain tensor ɛ_*ij*_ in terms of the sample surface coordinate system [see Fig. 1 ▸(*a*), *x* direction pointing into the sample] can be calculated according to ɛ_*q*_ = 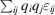 (Ramirez-Rico *et al.*, 2016 ▸). The scattering vector [see red arrow in Fig. 1 ▸(*a*)] is denoted **q** and has the components 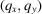 = 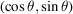. Here, θ is the Bragg angle for each reflection, determined from the average peak position in the *I* versus 2θ profile [see Fig. 2 ▸(*d*)]. Written in components this yields
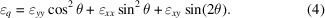
The projections of strain onto the three different scattering vectors of interest are given in Table 1 ▸ and the average over those projections is
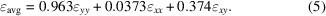
This is predominantly along the surface [*i.e.y* direction in Fig. 1 ▸(*b*)].

**Figure 7:**
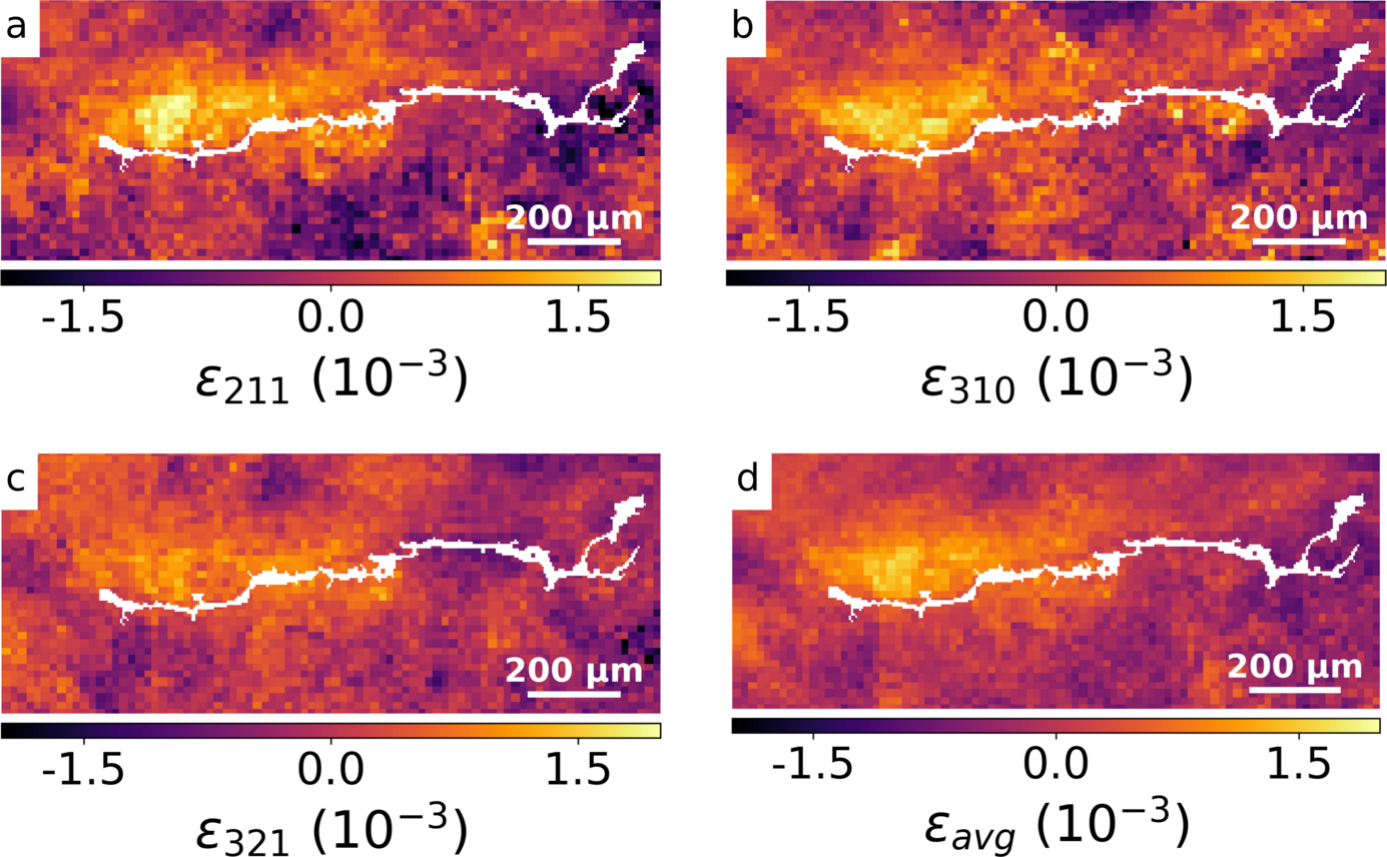
Relative strain projection maps with crack overlay for peaks (*a*) [211], (*b*) [310], (*c*) [321], and (*d*) displays the averaged strain map ɛ_avg_ of a shot-peened sample.

The strain projections from the selected peaks were first centered by subtracting their respective mean values. This was necessary since the relative accuracy of the strain-free lattice parameter *a*_0_ determined above was only 1.4 × 10^−3^, which is in the same order of magnitude as retrieved strain values. The as-determined strains constitute relative strains, *i.e.* strains with unknown offsets. However, strain differences within individual maps are quantitatively correct. The three selected peaks were then combined by algebraic averaging (*i.e.* equal contribution from each peak). The contributing strain projections and the resulting average strain ɛ_avg_ are shown in Fig. 7 ▸. The approach of combining information from several peaks is somewhat justified by the apparent improved homogeneity in Fig. 7 ▸(*d*) compared with Figs. 7 ▸(*a*)–(*c*).

**Figure 8:**
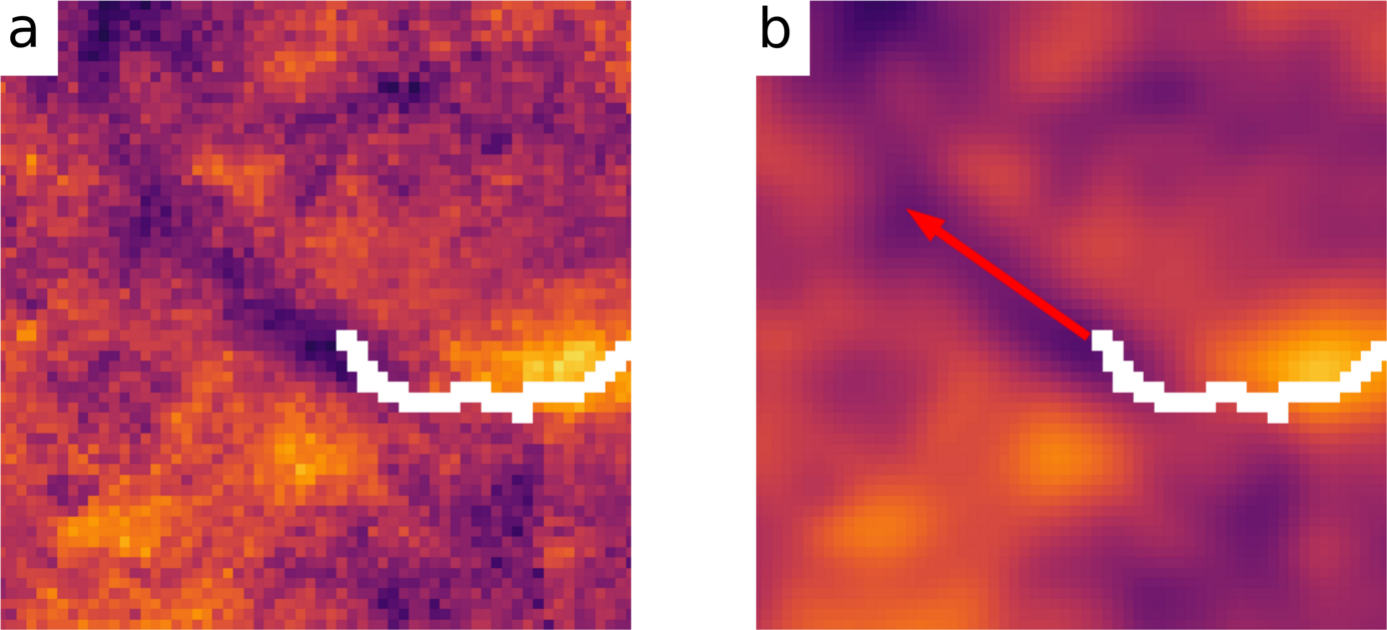
Heuristic prediction of crack propagation. (*a*) Zoomed-in view of Fig. 7 ▸(*d*). (*b*) Predicted path of crack propagation overlaid on a blurred version of (*a*).

Fig. 8 ▸(*a*) shows a zoomed-in view of the crack tip of the retrieved relative average strain shown in Fig. 7 ▸(*d*). A valley of relative strains in front of the crack is clearly visible and true compressive strain (as a special case of a strain valley in relative strains) may have been detected due to shot-peening of this sample. In LEFM further crack growth is in the direction of tensile strain in front of the crack tip. Taking into account the opposite sign in our study, this may suggest a heuristic for the further crack propagation estimated from experimental data: continued crack growth is expected in the direction of the strain valley. Fig. 8 ▸(*b*) shows the corresponding prediction.

**Figure 9:**
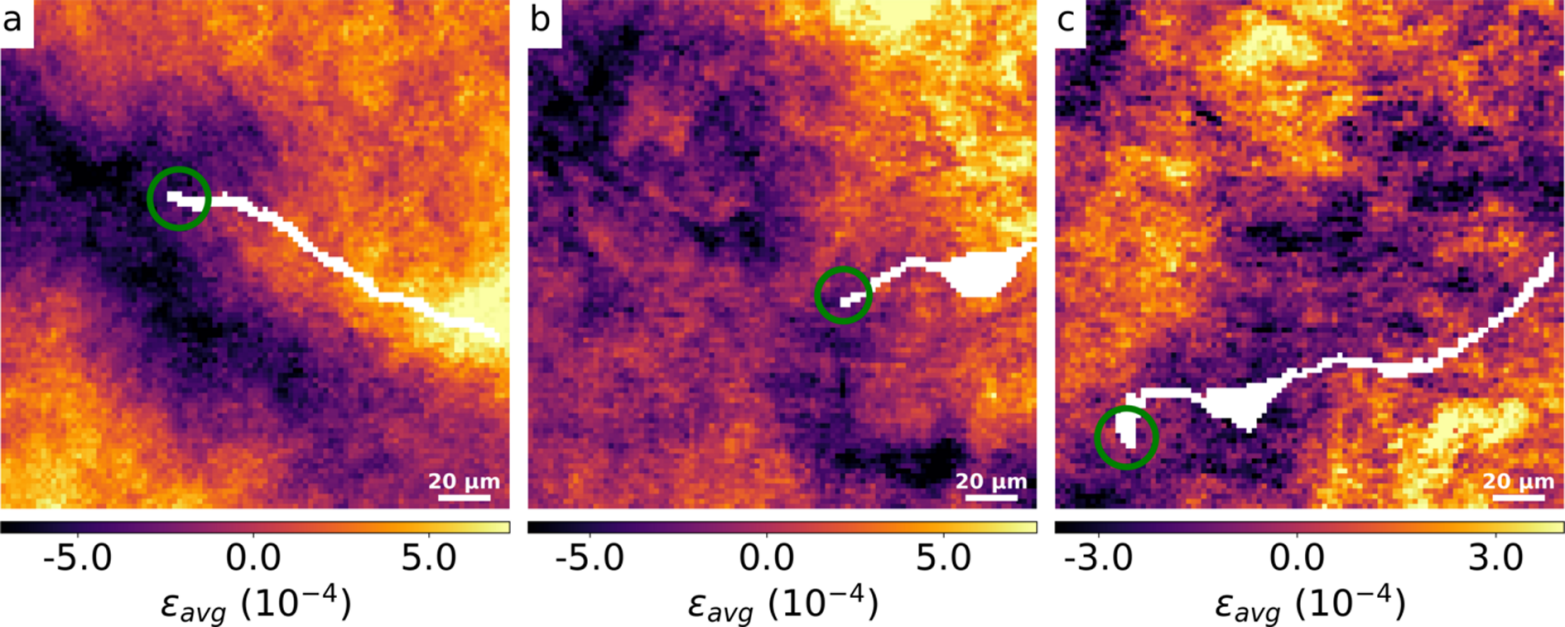
ɛ_avg_ maps from the crack tip for three different samples: (*a*) and (*b*) are shot-peened, while (*c*) is unpeened.

To determine the strain field around the crack tip rather than along the crack path, high-resolution scans with a step size of 1 µm were conducted in the crack tip region. Following the same procedure applied to the above scan, the resulting ɛ_avg_ maps for three distinct samples are depicted in Figs. 9 ▸(*a*)–9 ▸(*c*). Fig. 9 ▸(*a*) corresponds to the sample previously analyzed in the overview scan and data analysis sections, which is shot-peened, while Fig. 9 ▸(*b*) and Fig. 9 ▸(*c*) illustrate the results for two additional samples, with (*b*) being shot-peened and (*c*) being unpeened.

## 4. Discussion

Initial analysis revealed the effects of dynamic crystal recovery close to the crack (see Fig. 5 ▸), confirming that at least some of the XRD information originated in the vicinity of the crack. Furthermore, the initial overview scan revealed the strain distribution to be predominantly in the crack growth direction [see Fig. 1 ▸(*a*) and equation (5)] with a sensitivity of about *u*(ɛ_*q*_) = 1.5 × 10^−5^. The approach of combining information from several peaks is justified by the improved homogeneity in Fig. 7 ▸(*d*) compared with Figs. 7 ▸(*a*)–7 ▸(*c*). However, both the heterogeneity in the individual strain maps as well as the tortuous rather than line shape of the crack indicate mesoscale influences, such as type 2 stresses and/or grain orientation (Martinez & Hug, 2019 ▸; Withers & Bhadeshia, 2001 ▸).

Subsequent high-resolution scans focused at the crack tip offered a detailed view of the localized strain behavior. The strain field at the crack tip reveals a significant departure from the predictions of LEFM. LEFM anticipates strain singularities at crack tips, where strain magnitudes theoretically approach infinity under idealized conditions (Anderson & Anderson, 2005 ▸). However, the ɛ_avg_ strain maps from the crack tip region [see Figs. 9 ▸(*a*)–9 ▸(*c*)] indicate that such singularities are mitigated in practice.

Fig. 9 ▸(*a*), corresponding to the sample from the overview scan, and Fig. 9 ▸(*b*), representing another shot-peened sample, both show increasing strain values starting from the crack tip going along the crack path. Unfortunately, the crack tip for the unpeened sample [Fig. 9 ▸(*c*)] was just at the edge of the field of view, which precludes a substantial discussion. In addition, further crack growth may be in the vertical direction, which effectively switches the strains in crack growth and opening direction. This underlines the need for acquiring the two orthogonal strain directions simultaneously in future experiments.

Furthermore, *ex situ* TEM or HRTEM could offer atomic-scale strain distribution, but were excluded due to the destructive sample preparation (focused ion beam thinning < 100 nm) which alters macroscopic residual strains (Hÿtch & Minor, 2014 ▸). These techniques examine micro-residual strains, complementary to our XRD results, and would be beneficial for a future extension.

## 5. Conclusion

In this study, we employed scanning XRD of martensitic steel samples, including overview scans and scans targeted at the crack tip, to determine the strain field around the crack tip with micrometre spatial resolution. We have used co-registration of high-resolution optical microscopy and XRF images to pinpoint the crack position in the XRD contrasts. Further, we have identified dynamic recovery close to the crack using peak widths, which provided evidence for the claim that the XRD signal originates from the vicinity of the crack. We have used algebraic averaging over three peaks to calculate the average strain ɛ_avg_, improving visual homogeneity of the strain field. In terms of strain field distribution around the crack tip, we have demonstrated general congruence with LEFM. However, we also showed deviations, which are likely due to the complex mesoscopic structure of martensite. In conclusion, we have demonstrated that the multi-modal combination of XRD, XRF and optical microscopy constitutes a valuable tool for investigating strain fields around crack tips.

## Figures and Tables

**Table 1:** Strain projection for each *hkl* peak and the corresponding average used for equation (5)

[*hkl*]	θ (°)	ɛ_*q*_
[211]	8.70	0.977 ɛ_*yy*_ + 0.023 ɛ_*xx*_ + 0.299 ɛ_*xy*_
[310]	11.26	0.962 ɛ_*yy*_ + 0.038 ɛ_*xx*_ + 0.383 ɛ_*xy*_
[321]	13.36	0.946 ɛ_*yy*_ + 0.054 ɛ_*xx*_ + 0.449 ɛ_*xy*_
Average	–	0.963 ɛ_*yy*_ + 0.038 ɛ_*xx*_ + 0.377 ɛ_*xy*_
